# Attitudes Towards COVID-19 Vaccine: A Survey of Health Care Workers in Oman

**DOI:** 10.1007/s44197-021-00018-0

**Published:** 2021-12-20

**Authors:** Faryal Khamis, Abdallah Badahdah, Nawal Al Mahyijari, Furqan Al Lawati, Jaleela Al Noamani, Issa Al Salmi, Maher Al Bahrani

**Affiliations:** 1 Department of Medicine, Royal Hospital, PC 111 Aseeb, PO Box 1331, Muscat, Oman; 2 Department of Sociology and Rural Studies, South Dakota State University, Brookings, SD USA; 3 Psychosomatic Psychiatry, Sultan Qaboos Comprehensive Cancer Care & Research Center, Muscat, Oman; 4 Department of Psychiatry, Al Masarra Hospital, Muscat, Oman; 5 Department of Nursing, Royal Hospital, Muscat, Oman; 6 Department of Anesthesia and Critical Care, Royal Hospital, Muscat, Oman

**Keywords:** COVID-19, Vaccine, Health care workers, Oman, Hesitancy

## Abstract

Coronavirus Disease 2019 (COVID-19) vaccine hesitancy among health care workers (HCWs) is widely reported. Here we report on the prevalence of vaccine hesitancy and the factors associated with it in a sample of non-vaccinated HCWs. Data from 433 not vaccinated medical and non-medical HCWs from various health care facilities after the introduction of COVID-19 vaccination in Oman were analyzed. Most of the participants were nurses (41.5%) followed by physicians (37.5%) and non-medical HCWs (21%). Forty percent of HCWs were willing to uptake the COVID-19 vaccines. Physicians and male HCWs had more positive attitudes toward the COVID-19 vaccines than nurses and female HCWs. Concerns about the COVID-19 vaccines including unknown health issues, efficacy and safety were stated by the participants. Our results show a low level of willingness to uptake the COVID-19 vaccines among HCWs, an issue that must be urgently addressed.

## Introduction

Unwillingness to uptake COVID-19 vaccines has been reported in many countries around the world and among both the public and health care professionals. In both populations, reasons for rejecting or delaying vaccine uptake include concerns about vaccine safety, skepticism about the speed of vaccine development, potential sides effects, beliefs in conspiracy theories, and pre-existing health conditions [1–4].

HCWs play a critical role in vaccine promotion, thus expression of negative attitudes toward the COVID-19 vaccines might impact the success of COVID-19 national vaccination efforts [1, 4]. Research on COVID-19 vaccine hesitancy among HCWs has been reported worldwide [5]. Few studies, however, has been conducted from the public and HCW’s in the Arab countries of the Middle East and North Africa (MENA) region [6–8]. Studies among HCWs from this region identified several key reasons for refusing to uptake vaccines including possible sides effects, lack of sufficient data on the safety of the vaccines, and speed of vaccines development [9].

The overall goal of the current study was to identify the prevalence of COVID-19 vaccine hesitancy and factors associated with acceptance of vaccine uptake in a sample of non-vaccinated HCWs in Oman.

## Materials and Methods

### Study Design and Participants

We conducted an anonymous web-based questionnaire survey from several health care facilities from January 2021 to February 2021 to examine the impact of COVID-19 on HCWs in Oman. Participants received an invitation to participate in the study via hospital emails and the WhatsApp communication platform. In addition, the participants were asked to pass the invitation to their colleagues. We obtained informed consent from all participants prior to their participation. Only by reading the consent and accepting to participate, they could proceed to answer the survey questionnaire. We included all individuals aged 18 years or older, working in any health care facility and currently living in Oman.

A total of 680 responses from HCWs medical and non-medical (such as laboratory technicians, pharmacists, and administrators) were obtained during the study duration through QuestionPro platform. For the purpose of the current study, we excluded 247 HCWs who were vaccinated. The final analysis was carried out on 433 non-vaccinated respondents.

Ethical approval to conduct this study was granted by the Royal Hospital Research Ethics Committee in Oman (SRC#6/2021).

### Measures

Some of the items we used in this study were modified from previous studies on COVID-19 vaccine hesitancy [1, 4]. The questionnaire consisted of two sections.

#### Section 1: Sociodemographic, Experience with COVID-19 and Willingness to get Vaccinated

In the first part of the questionnaire, the participants were asked to provide some sociodemographic characteristics including: gender, age, marital status and whether they cared for COVID-19 patients. Furthermore, the were asked if they know a family member, or a colleague tested positive for COVID-19. These questions followed by the response options “yes,” “no,” and “not sure.” Next, we asked whether they would be willing to get vaccinated if the opportunity was offered to them on the day of the survey. The response was rated on a 5-point Likert scale that ranged from “strongly disagree” to “strongly agree.” A higher score indicates more willingness to get vaccinated.

#### Section 2: Attitudes Toward COVID-19 Vaccine

In this section we asked the respondents their perceptions of the COVID-19 vaccines using 11 supportive and positive statements toward the vaccine (e.g., all health care workers should be vaccinated against COVID-19) as well as concerns and negative statements (e.g., I think the COVID-19 vaccine might cause unknown serious health problems). All the items were rated using a 5-point Likert scale that ranged from “strongly disagree” to “strongly agree.” A principal component analysis with the Promax procedure (*k* = 4) showed that the 11 items accounted for 60% of the variance in the data. The Cronbach’s alpha coefficient was 0.93. A composite measure was created by taking the average of all items with high scores signifying more positive attitudes toward the COVID-19 vaccines.

### Statistical Analysis

Demographic information for the sample and the study variables were described using means and standard deviations or frequencies and percentages. For the ease of presentation, we grouped the 5-point Likert scale responses into three response categories: agree, disagree and unsure categories. That is, strongly agree and agree responses grouped in one category, strongly disagree and disagree responses in another category and unsure responses was treated as the third category.

A one-way ANOVA was used to compare groups. A statistical significance was based on *p* value of 0.05. All analyses were conducted with the statistical package for the social sciences (SPSS 27) software.

## Results

We analyzed data from 433 HCWs who were not vaccinated at the time of data collection. Among the participants, 41.5% were nurses, 37.5% were physicians and 21% were non-clinical staff. The age of the participants ranged from 23 to 72 years (*M*_age_ = 39.89, *SD* = 8.82). About 68.6% were females, 84.6% were married and 58% were non-Omani citizens. The distribution of gender by nationality showed that 76.3% of the Omani participants were females, whereas 63.3% of non-Omani were females.

About 38% of the participants had a family member who tested positive for COVID-19 and 90.5% knew a colleague/friend who also tested positive. The analysis by nationality showed that more Omani than non-Omani had a family member tested positive for COVID-19 (58.5% vs. 23.3%, *p* < 0.05). No difference was observed between Omani and non-Omani regarding knowing a colleague/friend who tested positive for COVID-19 (*p* = 0.21).

Among of the participants, 24.3% (*n* = 106) reported that they tested positive for COVID-19 with no significant difference by nationality (*p* = 0.50). Of those who tested positive, 26.42% were physicians, 60.38% were nurses and 13.59% were the non-medical HCWs. The proportion of Omani physicians and nurses who cared for COVID-19 patients was smaller than the proportion of non-Omani counterparts (35.7% vs. 64.3%, *p* < 0.05). Table 1 describes the *sociodemographic characteristics of participants by nationality.*

**Table 1:** Sociodemographic characteristics of participants by nationality

Characteristics	Nationality					Total
	Omani		Non-Omani			*n*
	*n*	%	*n*	%		
Age						
23–29	32	19.8	7		3.2	39
30–39	72	44.4	94		42.5	166
40–49	42	25.9	75		33.9	117
50–59	13	8	39		17.6	52
60+	3	1.9	6		2.7	9
Gender						
Females	142	76.3	162		63	304
Males	44	23.7	95		37	139
Occupation						
Physicians	72	38.7	94		36.6	166
Nurses	70	37.6	114		44.4	184
Others	44	23.7	19.1		19.1	93
Physicians and nurses who cared for COVID-19 patient	89	37.1	151		62.9	240
COVID-19 positive status						
Self	42	22.7	64		25.3	106
Know someone	174	93.5	226		88.3	400
Family member	103	57.2	57		22.5	160
Willingness to get vaccinated	75	40.3	101		39.6	176

The total might not tally because of missing data

**Fig. 1:**
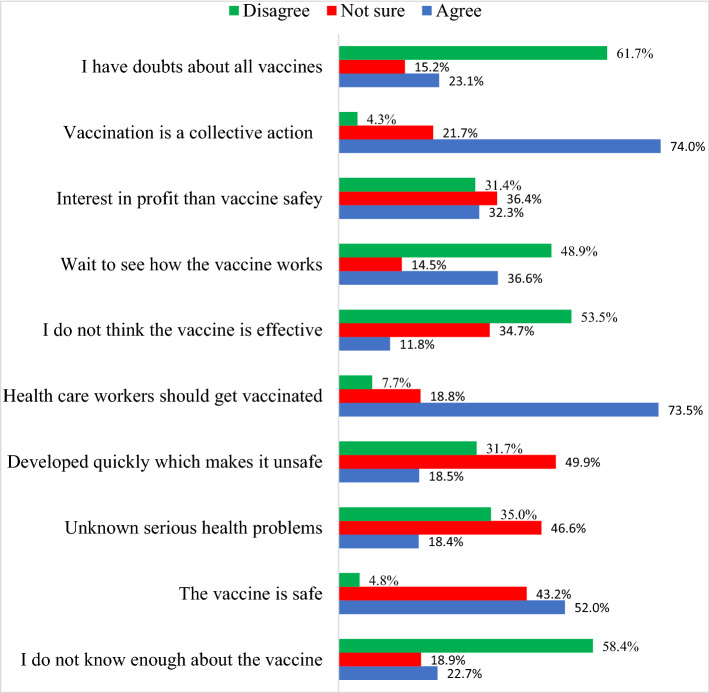
Percentage of HCWs who agreed, disagreed or unsure with the attitudes items

Among the 433 participants, 176 (40%) respondents were willing to have the COVID-19 vaccine if it was provided to them on the day of the survey, while 115 (26.1%) were not willing to be vaccinated and 150 (34%) were not sure. An ANOVA test showed no significant difference on the willingness to accept the vaccine by gender [*F* (1) = 2.85, *p* = 0.09], occupation [*F* (1) = 0.40, *p* = 0.67], or nationality [*F* (1) = 1.41, *p* = 0.24]. Figure 1 shows the willingness of the participants to get the vaccine.

Table 2 shows that the attitudes scale mean score was 3.47 (*SD* = 0.80). The means of all the 11 items were close to the overall scale mean expect three items. The means of “All health care workers should get the COVID-19 vaccine” and “Taken the COVID-19 vaccine is a collective action to prevent the spread of COVID-19” were above the scale mean (4.01 and 4.04, respectively), an indication of a general support for vaccination. On the other hand, the lowest mean (2.96) was for the item about the interest of pharmaceutical industry in making profit from the COVID-19 vaccines rather than the safety of the vaccines.

**Table 2:** Means and standard deviations for the attitudes items

Item	*M*	*SD*
1.I have concerned about the possible side effects of the COVID-19 vaccine	3.33	1.20
2.I will not get vaccinated, because I do not know enough about the COVID-19 vaccine	3.48	1.16
3.I am confident that the COVID-19 vaccine is safe	3.63	0.86
4.I think the COVID-19 vaccine might cause unknown serious health problems	3.21	0.96
5.The research and development process that produces the COVID-19 vaccine was quick which makes the vaccine unsafe	3.15	0.90
6.All health care workers should get the COVID-19 vaccine	4.01	0.96
7.I do not think that the COVID-19 vaccine is effective	3.61	0.99
8.I will wait to see how the COVID-19 vaccine works for others before I get vaccinated	3.16	1.26
9.The pharmaceutical industry is more interested in profit than making sure that the COVID-19 vaccine is safe	2.96	1.11
10.Taken the COVID-19 vaccine is a collective action to prevent the spread of COVID-19	4.04	0.91
11.In general, I have doubts about all kinds of vaccines including the COVID-19 vaccine	3.55	1.20

An ANOVA test showed male HCWs had (*M* = 3.64, *SD* = 0.80) more positive attitudes toward the vaccine than females (*M* = 3.39, *SD* = 0.78); [*F* (1) = 5.95, *p* < 0.05]. There were no differences between Omani and non-Omani physicians [*F* (1) = 0.28, *p* = 0.60] nor between non-Omani and Omani nurses in their attitudes toward the COVID-19 vaccines [*F* (1) = 2.40, *p* = 0.12]. However, the Omani non-medical staff expressed more negative attitudes than their counterparts [*F* (1) = 7.41, *p* < 0.05].

## Discussion

This study was conducted during the early phases of COVID-19 vaccine rollout. The acceptance rate of COVID-19 vaccine among healthcare workers during the study period was 40%. The acceptance rate among physicians and male HCWs was higher than nurses and female HCWs. The low acceptance rate was observed despite the significant psychological impact of the COVID-19 pandemic on HCWs in Oman, as shown in a recent study, where there was a high prevalence of stress, anxiety, and poor psychological well-being, especially among females, young health care workers and those who interacted with known or suspected COVID-19 patients [10]. In another study, nurses, Omani nationals, and frontline HCWs were the most impacted by the global health crisis. Moreover, HCWs who cared for COVID-19 patients reported a higher stress level, lower level of well-being, and bad sleep quality more so in Omani than non-Omani [11].

Several vaccines have been approved for use in the Gulf Cooperate Council Countries, nevertheless vaccine hesitancy is widespread among both the public and HCWs in the region [6–9]. Concerns about the COVID-19 vaccines safety, efficacy, and adverse health outcomes were among the determinants of vaccine hesitancy [6, 9]. In addition, the accelerated pace of vaccine development has further heightened public anxieties and affected acceptance [12]. In our study, these factors can be illustrated by the percentage of HCWs who endorsed some of the attitude’s items. For example, four in ten said they were not sure about the vaccine safety and that the speed of the vaccine development might make it unsafe. A similar proportion of HCWs was concerned about possible future health problems associated with the vaccines. Furthermore, one-third questioned the efficiency of the vaccines. On the positive side, seven in ten agreed that vaccination is a shared responsibility and that all HCWs should be vaccinated.

In the present study, only 40% of HCWs agreed or strongly agreed that they would get a vaccine for the COVID-19 if it was offered to them on the day of the survey. Moreover, six in ten HCWs were either opposed or not sure if they would take the vaccine. This suggests that some HCWs in our cohort lack the knowledge on vaccine-preventable diseases. Research shows that vaccine acceptance among HCWs increases proportionately with their level of training on this topic [13, 14]. Therefore, focusing interventions that address the general knowledge of HCW’s on the process of vaccine production and approvals for efficiency and safety trials are inevitable to optimize the vaccine acceptance among HCW’s in this region.

In this study, the acceptance rate of COVID-19 vaccination was comparable to some studies from the globe and the region that were conducted during or just before the initial vaccine rollout. For example, the acceptance rate among HCWs was 52.52% in Saudi Arabia, SA (December 2020) [9], and 48.6% in France and French speaking Belgium and Quebec (October–November 2020) [2]. These acceptance rates are higher than the 33.2% from SA (December–January 2020) [15], 24.5 to 36% from USA (September–October 2020) [16] and 27.7% from the Democratic Republic of Congo (March–April 2020) [14] but lower than the 64.9% reported acceptance rate from SA (October–November 2020) [17], 60–90% among physicians in Greece (February 2020) and 60% from the nurses in Hong Kong, China (February–March 2020) [18].

Similar to other studies [9, 13–18], male HCWs had more positive attitudes than female HCWs and physicians had more positive attitudes toward the COVID-19 vaccines compared to nurses [9, 13–17]. This result is mystifying considering that more nurses in this study tested positive for COVID-19 (35.4%) compared to physicians (17.1%). However, this puzzle might be explained by a recent data that showed although the rate of infection among women and nurses was higher than men and physicians, the rate of death was higher among men and physicians than women and nurses [19]. In addition, risk perception is strategic for vaccine decision-making. It is possible that the perceived risk could have been lower in our cohort due to the relatively low number of confirmed cases and deaths at the time of conducting this study. On the other hand, many female HCWs were at childbearing age, and fear of vaccination side effects on future pregnancies might have played a role in vaccination hesitancy in this group. Furthermore, women tend to adopt more to the non-pharmacological interventions such as hand washing, sanitization and masking [14].

In this study, non-Omani non-medical HCWs had more positive attitudes toward the COVID-19 vaccine than their Omani counterparts. Similarly, several studies from SA have showed that expatriates were more willing for the vaccination [15, 17]. This could be due to their limited social circle of interaction and not being influenced by the negative perception on vaccination from their relatives and friends. In addition, non-Omani non-medical HCWs might have higher trust in the health system. Numerous studies reported that a higher trust in the health system can predict vaccination acceptance [5].

The study has few limitations that warrant consideration. The data were collected using online self-report questionnaire which might be susceptible to the effect of social desirability. In addition, the research was conducted early into the vaccine rollout, where vaccine safety data were not widely available for review. Another limitation is the utilization of one item to gauge the participants’ willingness to uptake the vaccine. Hence, future studies should use a composite measure based on a theoretical framework such as the theory of planned behavior.

## Conclusion

COVID-19 vaccine hesitancy remains high among HCW’s in Oman even after the introduction of the vaccines. Identifying the barriers to vaccination and demographic characteristics of the HCW’s who refuse vaccination is essential. Interventions targeted towards increasing acceptance rates among HCW’s are urgently needed.
